# Effect of short-term educational intervention on complementary feeding index among infants in rural Bangladesh: a randomized control trial

**DOI:** 10.1186/s40795-022-00565-0

**Published:** 2022-08-02

**Authors:** Aminur Rahman, Mohammad Badrul Bhuiyan, Sumon Kumar Das

**Affiliations:** 1 Health System and Population Studies Division, International Centre for Diarrhoeal Disease Research, Bangladesh (icddr,b), Dhaka, Bangladesh; 2 Liverpool School of Tropical Medicine (LSTM), Liverpool, UK; 3 Menzies School of Health Research, Northern Territory, Australia

**Keywords:** Infants, Stunting, Underweight, Malnutrition, CF, Infant feeding, Rural, Bangladesh

## Abstract

**Background:**

Timely, adequate and appropriate Complementary Feeding (CF) is essential for the growth and cognitive development of infants, but until today, evidence-based information is scarce in terms of impact evaluation of CF index (CFI). The study aimed to examine the effect of the short-term intervention of promoting CF practices on the nutritional status of infants in rural Bangladesh.

**Methods:**

An educational-intervention study followed a randomized controlled trial (RCT) design (NCT03024710). Mothers and family members in the intervention arm received intensive counselling on CF through community health workers (CHWs), whereas existing healthcare services were received in the comparison arm. The study was carried out in the rural Matlab sub-district of Bangladesh between April 2011 and March 2013. In the specified study areas among 360 mother-infant pairs systematically assigned into intervention group and comparison group. Short-term educational intervention on CF was provided for the intervention group and existing services were un-intervened for the comparison group. The outcome of interventions was evaluated after the implementation period using Generalized equation estimation model.

**Results:**

At baseline, the study participants were not different except mean height (*p* = 0.04), weight-for-age Z score (WAZ) (*p* = 0.03) and religion (*p* = 0.04) in between two groups. The mean CFI was significantly higher at intervention area than the comparison and higher category of CFI (score 10 or more) was significantly higher at intervention area than comparison. After adjustment, one-unit CFI increased height-for-age z score by 0.07 units and decreased WAZ by 0.13 units in the intervention group but not significantly changed observed at comparison group.

**Conclusion:**

Guided short-term nutritional intervention and developed CFI indicated a significantly better score in intervention area than comparison groups and would be a well adaptable tool for future studies.

**Trial registration:**

The trial was registered (NCT03024710) at clinical trial registration website. Date of registration: 1/19/2017.

**Name of the registry:** Clinical Trial.gov.

**Date of registration:** 19/1/2017 (retrospective registered).

**URL of trial:** https://clinicaltrials.gov/ct2/show/NCT03024710

## Background

Globally 165 million under-five years children are suffering from chronic undernutrition and 90% of them live in only 36 countries [1]. Poor nutrition increases the risk of childhood morbidity, responsible for one-third of under-five year’s deaths [1]. Thus, the evidence-based benefits of breastfeeding and optimal complementary feeding (CF refers to the introduction of safe and nutritional foods in addition to breastfeeding at about six months of age) for child’s survival, growth and development have been well adopted irrespective of the burden of childhood malnutrition [2–4]. According to the World Health Organization (WHO), CF should be given timely, appropriately and in sufficient quantity [4]. The period of CF (from 6 to 24 months) is one of the most critical times [5, 6] when maximum growth faltering happens and it is challenging to reverse the process specially stunting and some other functional deficits [7, 8]. Exclusive breastfeeding (EBF) could prevent 13% of child deaths,while appropriate CF practices are attributable to a further 6% reduction of global under-five year mortality [9]. However, there is a lack of efforts to increase the knowledge of good CF practices at the community level in developing countries, including Bangladesh.

Inappropriate practices of CF reflect the knowledge gap at the population level resulting in undernutrition [10]. Thus, a comprehensive programme of integrated actions on many fronts has been addressed, including promoting improved infant and young child feeding (IYCF) practices. At the community level, mothers and families (along with peer groups) need support to initiate and sustain appropriate IYCF practices [11]. In a different context, educational intervention on CF practices showed notable nutritional improvement [12–14]. However, they differ from duration of EBF and starting of CF practices as recommended by WHO [15]; and none of them evaluated feeding practices through CF index (CFI; a composite score that reflects the quality of implementing any nutritional intervention and helps to identify which varieties of recommended meal/s is/are not taken by infants or children). A few studies in Bangladesh assessed CFI [16–18] but scarcity in following the WHO guidelines on IYCF by using six standard indices of CFI measurement [15]. Recently, a few cross-sectional studies determined the association of CFI with childhood undernutrition; but the determinants mostly remained unpredictable, mainly due to the lack of a strong study design such as randomized control trial (RCT) [19, 20]. Therefore, the present community-based RCT was conducted to examine the effect of the short-term intervention of CF promotion (through CFI) on the nutritional benefits of infants in rural Bangladesh.

## Methods

### Study site and participants

The study was conducted in the rural community of Matlab sub-district, Chandpur, Bangladesh, located 56 kms southeast of Dhaka, the capital city of Bangladesh. The International Centre for Diarrhoeal Disease Research, Bangladesh (icddr,b) has been conducting a Health and Demographic Surveillance System (HDSS) in a population of about 220,000 since 1966. At Matlab, a comprehensive maternal, neonatal and child health (MNCH) programme was initiated in March 2007. Under this programme, all pregnancies in the area are being identified and followed-up bi-monthly at the household level. In addition, two additional visits are also being done (at 12–14 weeks and at 33–34 weeks of pregnancy) and counselling are also being provided to the mothers about facility-based delivery, antenatal care, and birth preparedness [21]. For the present study, infants who completed six months of age were selected along with their mothers from the MNCH database. This database was upgraded bi-weekly basis after receiving information from Community Health Research Workers (CHRWs) and cross-checked by the field supervisors.

### Study design and randomization

The study followed an RCT design. The MNCH programme area was divided into four blocks and each one comprises of an approximate 27,000 population [18]. An independent researcher (who was not involved in the study in any way) paired the blocks into two, where each pair was assigned randomly into two groups: (i) the intervention group and (ii) the comparison group. To avoid information contamination, four blocks were paired into A, B and C, D according to the geographical location. Block C and D were selected as intervention, and A and B were comprised as comparison areas.

### Sample size and enrolment

**Fig. 1:**
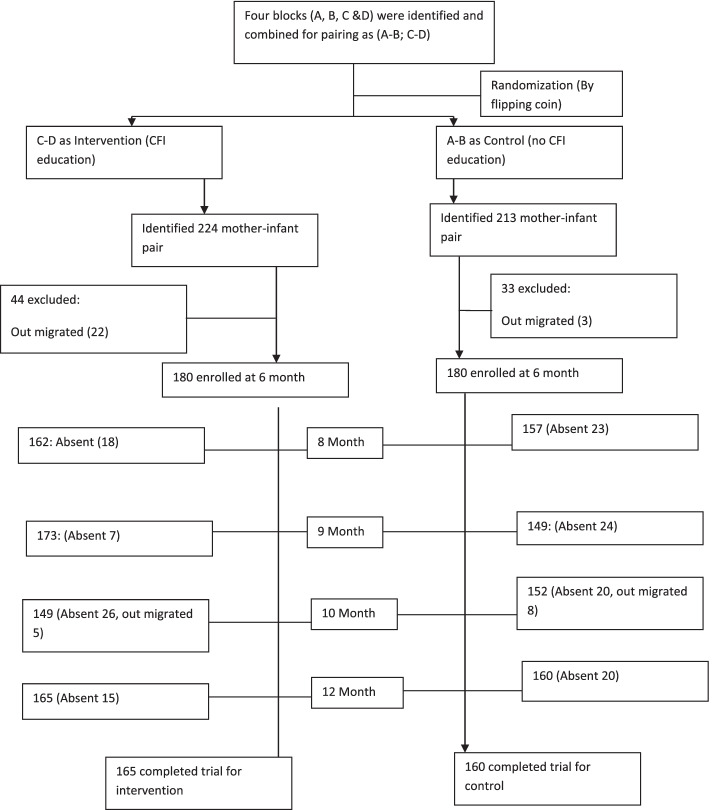
Trial profile

The required sample was calculated based on earlier studies [17]. Assuming the intervention effect could reduce stunting at 20% with an available 41% of prevalence [22]. With a 5% significance level and 80% power, a design effect of 1.5 and a maximum of 30% dropout, the expected sample size would be 180 infant-mother pairs in each arm (Fig. 1).

### Intervention

At six months, all enrolled children and their mothers (around 180 child-mother pairs) and a close companion were invited to the sub-centre for a session to deliver a set of standard information. These were: washing hands every time before preparing a meal for the infant, frequency of meal and the quantity of feed according to the age, consistency of meal, diversity of food for every day and responsive feeding. The Principal Investigator (PI) took one and half hours to conduct each session. After the end of the sharing of information, the CHRWs measured the anthropometric indices (weight and height) following standard measuring tools and procedures in the presence of the PI and study supervisor. Similar refresher sessions were carried out at nine months. In addition, the CHRWS visited every selected child-home during 8, 10 and 12 months, delivered similar information, took the anthropometric measurements and recorded accordingly. Variables considered under the CFI measure have been discussed in Table 1.

**Table 1:** Variables consider under dependent and independent variables

Dependent	Independent
HAZ (Height-for-age Z score)	Mother Age
WAZ (Weight-for-age Z score)	Education
WHZ (Weight-for-height Z score)	
Stunting (HAZ < -2SD)	Birth order
Underweight (WAZ < -2SD)	Child sex
Wasting (WHZ < -2SD)	Socio-economic status
CF Index (variables)	Religion
● Breastfeeding	
● Bottle feeding	
● Initiation of CF	
● Dietary diversity (last 24 h)	
● Food group frequency (Past 7 days)	
● Meal frequency (last 24 h)	

### Implementation

Mothers and infants of completed six months were randomly identified from the MNCH database for both areas between March 2011 and June 2011. In the beginning, baseline information was collected for each participant. If an infant was absent during an initial household visit a schedule reattempted was carried on. In addition, an infant with severe illnesses or handicaps which affect development, feeding, or activity and was absent during enrolment was excluded. The enrolment details have been described in Fig. 1 (Trial profile).

All enrolled mothers and other family members received two training (at enrollment and at nine months) on standard CF practices at designated sub-centre health facilities for the intervention area. A training manual was developed following WHO and Bangladesh Breast Feeding Foundation guidelines.

### Ethical approval

An institutional review board (IRB) of icddr,b reviewed the protocol and provided approval. The protocol was registered (NCT03024710) on the clinical trial registration website.

### Data collection and quality control

Trained community health research workers (CHRW), Field Research Assistants (FRAs) and Field Research Supervisors (FRSs) were assigned to monitor all the study activities. Children’s weight and length (both intervention and comparison groups) were measured during enrolment at 6th, 8th, 9th, 10th and 12th months of age following standardized methods and their weight-for-age (WAZ; underweight < -2SD), height-for-age (HAZ; stunting < -2SD) and weight-for-height (WAZ; wasting < -2SD) were estimated following WHO-Anthropac 2005 software. Field Research Assistants (FRAs) and Field Research Supervisors (FRSs) received training on study questionnaires and anthropometric measurements. The FRAs were responsible for all supervision and monitoring of data collection. Day-to-day supervision and monitoring was carried out by the FRSs using the SOP checklist. Any inconsistency or queries were re-assessed upon discussing with the respective CHRW and FRA. For quality control, 5% of participants were randomly chosen and were re-interviewed by the trained FRAs within two weeks after the routine interview.

### Complementary feeding index score

Table 2 describes the scoring pattern of six variables for CF practices of infants aged 6–12 months, such as continued breastfeeding, avoiding bottle feeding, timely initiation of CF, dietary diversity (past 24 h), food group frequency, meal frequency (past 24 h). The following seven food groups were consumed: starch staple (rice, kichuri, potato, roti, suzi etc.), pulses, milk (other than breast milk), meat/eggs, vit-A rich fruit, vegetables, other fruits and vegetables and others.

**Table 2:** Variables and scoring system used to construct the CFI

	**Scores**	
**Variables**	**6–8 months**	**9–12 months**
Breastfeeding	No = 0, Yes = 2	No = 0, Yes = 2
Bottle feeding	No = 0, Yes = 1	No = 0, Yes = 1
Initiation of CF	No = 0, Yes = 2	No = 0, Yes = 2
Dietary diversity (last 24 h)	Low (no diversity) = 0, Medium (1–2 diversity) = 1, High (≥ 3 diversity) = 2	Low (no diversity) = 0, Medium (1–3 diversity) = 1, High (≥ 4 diversity) = 2
Food group frequency (past 7 days)	Nil = 0, 1–2 food group = 1, ≥ 3 food group = 2	Nil = 0, 1–3 food group = 1, ≥ 4 food group = 2
Meal frequency (last 24 h)	No meal was given = 0, Only single meal given = 1, ≥ 2 meals given = 2	No meal was given = 0, 1–2 meals given = 1, ≥ 3 meals given = 2
**Total maximum CFI score**	**11**	**11**

The dietary diversity (last 24 h), food group (past seven days) and meal (last 24 h) frequency were categorized and scored following ICFI guideline [23]. The detail scorning for each ICFI items was given in Table 4.

Theoretically, the CFI score ranges from 3 to 11 (Table 2). Therefore, for analysis, we combined the two age-groups and grouped into terciles to form 3 categories of CF practices- low (7 or below), medium (8, 9), and high (10 or above).

### Co-variates

Several maternal socio-demographic data were collected. Such as asset quintile (poor, middle, rich); education of the mother (collected from HDSS database), age of the mother (calculated from the date of birth to the date of entry in the study and grouped a < 20 years, 20–24 years, 25–29 years, ≥ 30 years); religion (Muslim and others); sex of the infant; education of the mother [no education, up to primary (1–5 class), up to secondary (6–10 class) and above secondary (≥ 11 class)]; birth order (1, 2–3 and ≥ 4). To estimate asset quintile assets included durable goods (e.g., table, chair, watch, television, or bicycle), housing facilities (e.g., type of toilet, or source of drinking water), housing materials (e.g., type of wall or roof), and possession of farming land.

### Analysis plan and outcome measures

Baseline characteristics of the two groups were examined at enrollment. A Chi-square and t-test were performed to observe any association between the groups for categorical and continuous data, respectively. A *p*-value of < 0.05 was considered significant. The primary outcome measure was to assess the change in all CFI indices in three-time points at baseline (6 months), mid-time point (9 months) and at endline (12 months). The secondary outcomes were to assess the impact of CFI score (exposure) on changes in three nutritional outcomes (HAZ, WAZ and WHZ) over time. A Generalized Estimating Equation (GEE) was used separately for their nutritional indices for intervention and control group l [24]. We considered three separate models to understand the potential confounding and modifying effect. Model 1 only CFI; model 2 = Model-1 with child age and sex; and Model-3 = Model-2 with maternal education, and asset score and religion.

Data cleaning was carried out by the Statistical package for Social Sciences (SPSS, version 19) and per-protocol analyses were performed using Stata (version 13).

## Results

Detailed baseline characteristics of study participants were presented in Table 3 and found similar characteristics between the intervention and comparison groups (Table 3). Mother’s education, religion and child’s height and HAZ were found to be comparable between the two groups at baseline. A significantly higher proportion of mothers in the intervention group had no education than in the control group; most mothers from both intervention and control had secondary education. More than 80% of the study mothers were Muslim. On average, children from the intervention and control groups had 65 cm height during baseline and mean HAZ was higher in the intervention than in the control group.

**Table 3:** Baseline characteristics of the study participants at enrolment

Indicators	Intervention (*N* = 180)	Control (*N* = 180)	*p*-value/t-test
	n (%)/ mean (± SD)	n (%)/ mean (± SD)	
**Mother characteristics**			
**Age**	26.9 (± 0.5)	27.2 (± 0.5)	0.74
< 20 years	14 (7.8)	8 (4.4)	0.56
20–24 years	61 (33.9)	53 (29.4)	
25–29 years	51 (28.3)	43 (23.9)	
≥ 30 years	49 (27.2)	53 (29.4)	
**Education**			
No education	19 (10.6)	6 (3.3)	0.05
Up to primary (1–5 class)	52 (28.9)	52 (28.9)	
Up to secondary (6–10 class)	101 (56.1)	108 (60.0)	
Above secondary (≥ 11 class)	8 (4.4)	14 (7.8)	
**Birth order**			
1	67 (37.2)	68 (37.8)	0.86
2–3	93 (51.7)	89 (49.4)	
≥ 4	20 (11.1)	23 (12.8)	
**Child characteristics**			
**Sex**			
Male	84 (46.7)	91 (50.6)	0.46
Female	96 (53.3)	89 (49.4)	
**Height (cm)**	65.6 (± 0.2)	65.0 (± 0.2)	0.04
**Weight (kg)**	7.04 (± 0.1)	7.01 (± 0.1)	0.72
**Height for age z-score**	-0.48 (± 0.1)	-0.78 (± 0.1)	0.03
**Weight for age z-score**	-0.72 (± 0.1)	-0.79 (± 0.1)	0.56
**Height for weight z-score**	-0.46 (± 0.1)	-0.31 (± 0.1)	-0.23
**Household information**			
**Socioeconomic status**			
Poor	59 (32.8)	60 (33.3)	0.97
Middle	36 (20.0)	34 (18.9)	
Rich	85 (47.2)	86 (47.8)	
**Religion**			
Muslim	147 (81.7)	161 (89.4)	0.04
Others	33 (18.3)	19 (10.6)	

*SD* Standard deviation

**Fig. 2:**
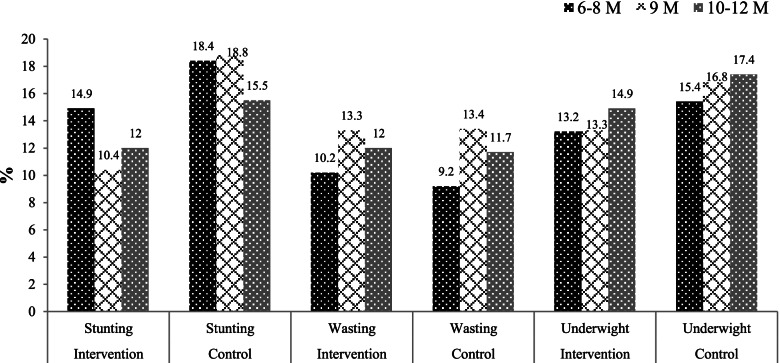
Proportion of nutritional indices both in intervention and control areas by different visit periods

The proportion of stunting and underweight were persistently lower in the intervention than the control group at all assessment time points. But there were no differences observed in wasting (Fig. 2).

The distribution of components considered to compute the CFI score at three time points six months, nine months and 12 months were shown in Table 4. Significant differences among the intervention and control groups were observed for bottle feeding at three different time points. During nine months and 12 months, more than two-thirds of intervention children had high dietary diversity and medium food group frequency than the control group, and the differences were statistically significant. However, meal frequency in intervention and control groups was statistically significantly different in 12 months only, where maximum babies had high meal frequency for both groups.

**Table 4:** Distribution of infant complimentary feeding index indicators

	Six months (Baseline)			Nine months			Twelve months (Endline)		
	Intervention (*n* = 180)	Control (*n* = 180)	*p*-value	Intervention (*n* = 173)	Control (*n* = 149)	*p*-value	Intervention (*n* = 160)	Control (*n* = 165)	*p*-value
**Continued breastfeeding**	180 (100)	177 (98.3)	0.08	173 (100)	146 (97.9)	0.06	160 (100)	161 (97.6)	0.05
**Not Bottle feeding**	150 (83.3)	130 (72.2)	0.01	156 (90.2)	117 (79.1)	0.01	146 (91.3)	132 (80)	0.00
**Initiation of complementary feeding**	160 (88.9)	152 (84.4)	0.17						
**Dietary diversity (past 24 h)**									
Low (no diversity)	21 (11.7)	29 (16.1)	0.46	2 (1.2)	1 (0.7)	0.00	0 (0)	1 (0.6)	0.00
Medium (1–2/1-3^a^ diversity)	103 (57.2)	100 (55.6)		45 (26)	86 (57.7)		50 (31.25)	83 (50.3)	
High (≥ 3/ ≥ 4^a^ diversity)	56 (31.1)	51 (28.3)		126 (72.8)	62 (41.6)		110 (68.8)	81 (49.1)	
**Food group frequency**									
Low (Nil)	24 (13.3)	40 (22.2)	0.06	0 (0)	11 (11.6)	0.00	0 (0)	5 (4.8)	0.03
Medium (1–2/1-3^a^ food group)	91 (50.6)	89 (49.4)		81 (62.3)	55 (57.9)		85 (69.1)	63 (60.6)	
High (≥ 3/ ≥ 4^a^ food group)	65 (36.1)	51 (28.3)		49 (37.7)	29 (30.5)		38 (30.9)	36 (34.6)	
**Meal frequency (last 24 h)**									
Low (no meal was given)	20 (11.1)	30 (16.7)		2 (1.2)	1 (0.7)		0(0)	1(0.6)	
Medium (Only single/ 1-2^a^ meal given)	18 (10)	12 (6.7)	0.20	5 (2.8)	10 (6.7)	0.25	2(1.3)	12 (7.3)	0.02
High (≥ 2/ ≥ 3^a^ meals given)	142 (78.9)	138 (76.7)		166 (96)	138 (92.6)		158(98.8)	152 (92.1)	
**CFI score**									
Mean (± SD)	8.71 (± 0.1)	8.16 (± 0.1)	0.01	9.96 (± 0.1)	9.16 (± 0.1)	0.00	9.91 (± 0.1)	9.46 (± 0.1)	0.00

^a^9-12 months

*SD* Standard deviation

**Fig. 3:**
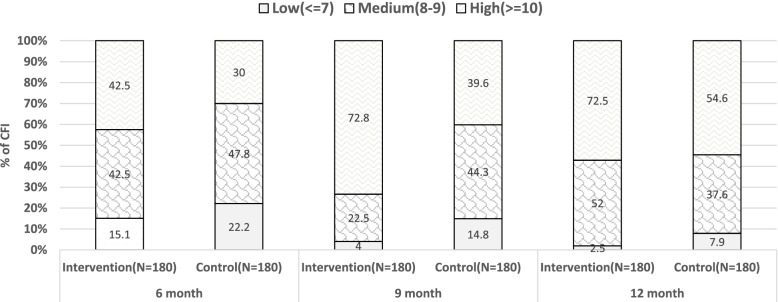
Proportion of Complimentary Feeding Index by categories by areas

The proportion of High CFI (> = 10) were persistently Higher in the intervention areas than control areas at all assessment time points (Fig. 3).

After adjusting for potential covariates, in the final model (Table 5; Model-3), the CFI score significantly impacted a change in HAZ and WHZ over time in the intervention group. In the intervention group, one unit change in CFI score changed the mean positive 0.07 units (increased) of child’s HAZ score; negative 0.13 (decreased) units of WHZ. However, no significant change was observed in the control group.

**Table 5:** Association between mean HAZ, WAZ, WHZ and CFI score through Generalized Estimating Equation (GEE) analysis

**CFI score**	**Intervention;** Coefficient (95% CI) p			**Control,** Coefficient (95% CI) p		
	**HAZ**	**WAZ**	**WHZ**	**HAZ**	**WAZ**	**WHZ**
**Model 1**	0.07 (0.01, -0.13) 0.02	-0.04 (-0.09, -0.02) 0.2	-0.13 (-0.21, -0.04) 0.00	0.04 (-0.09, 0.16) 0.56	-0.01 (-0.15, 0.12) 0.86	-0.02 (-0.23, 0.19) 0.87
**Model 2**	0.07 (0.01–0.13) 0.02	-0.04 (-0.09–0.02) 0.2	-0.13 (-0.21, -0.04) 0.00	0.04 (-0.08, 0.16) 0.55	-0.01 (-0.15, 0.12) 0.86	-0.02 (-0.23, 0.19) 0.88
**Model 3**	0.07 (0.01–0.13) 0.02	-0.04 (-0.09–0.02) 0.21	-0.13 (-0.21, -0.04) 0.00	0.04 (-0.10, 0.17) 0.59	-0.01 (-0.15, 0.13) 0.88	-0.02 (-0.23, 0.19) 0.87

Model-1: only CFI

Model-2 = Model-1 with child age and sex

Model-3 = Model-2 with maternal education, and asset score and religion

## Discussion

The present study was one of the unique educational intervention studies with a robust methodology indicated that the education improved the overall CFI index among the intervention children. Furthermore, this short-term CFI change also significantly impacted the change in HAZ scores and WAZ scores among intervention children than in comparison groups. The girls were less stunted and underweighted in the intervention area than the control (data not shown).

Considering different observation points (for example, between six, eight and nine months etc.) significant changes were observed between the study infants. The educational intervention on recommended food choice (dietary diversity, food groups which constitutes essential items and meal frequency for infant) for weaning infants are higher in intervention areas than in control which has demonstrated that mothers in the intervention area gained better knowledge about food choices which were crucial for child nutritional development during initiation of weaning foods. Moreover, the CFI is significantly high during a three-time period among infants in the intervention area than in the control area which specifies the caregivers have adequate quantity and quality knowledge on the infant feeding practices that might be made notable differences in changing the growth of infants in the intervention area reflected by less stunting and underweight. Active participation of other family members in educational sessions also might accelerate the infant's growth as most children at this weaning stage do not consume a balanced diet mainly due to a lack of knowledge of the mothers or caregivers. These findings are consistent with another study [25]. A previous study documented the importance of household food security in infant and young child feeding and child growth in a similar setting, but we did not aim to reassess food security among our current study population [26]. Furthermore, food hygiene and infection might be the other contributing factors for relatively better nutritional catch-up during later infancy.

Our study showed the proposed intervention for the girls had lower rates of underweight and stunting than boys, eliminating the sex inequity perspective if intervention strength and family’s positive perception, attitude and practices for infant’s of both sex. Thus, the CFI intervention has significant public health implications as well as policy messages. Such as, an early realization of nutritional importance for girls who would contribute equally in future country progress and to achieve the sustainable development’s goal [27], become a healthier mother who could deliver a healthy newborn and can contribute in national economic growth and it’s long-term sustainability.

A study conducted among the Bangladeshi population in the United Kingdom found CFI is important to measure the status of nutrition among children with the feeding practices, but the process is challenging to implement. The very small study sample size would be difficult to make a judgment about the outcome of CFI [28]. In addition, developing CFI other than RCT designs may not be methodically robust to use in a health system as carried out some epidemiological studies following other study design as reported in a recent systematic review [29].

The strength of this study is the computed CFI has come from an RCT design probably in its first, the other available CFI has been computed mostly from the survey or hospital follow up study. This could have been shaped better through individual randomization in future studies. But, even though a composite CFI score definitely has policy implications as through CFI score this is easy to understand which factors and areas are not in line with nutritional improvement to guide in evaluating a nutritional program and tell the program managers specifically which needs to be improved for better nutritional indices. This study's main strengths are the quality and robustness of this randomized control data. Furthermore, the rigor of the data quality procedures and strict follow-up like the study has provided a unique opportunity to produce authentic results (nearly no chances of information bias) from the analysis [30].

Like other RCTs the current study also had some limitations. The randomly selected intervention area is outside the Matlab sub-district township, and the comparison area is within the Matlab Township. This might dilute the relationship of nutritional indices with CFI as the town has an inbuilt priority to receive any public messages. In addition, during the study period, there was also a continuous national campaign on promotional messages of CF practices through mass media (radio and TV). This might contaminate the study area, but we think both the study areas had equal chances to getting that information. However, the study site, icddr,b service area, may not be ideal or not being representative of other rural areas for this type of educational evaluation because icddr,b has been conducting many short and long-term interventions in health, population and nutrition [31]. Moreover, due to the non-flexibility of the data available, we might be missing some essential contextual variables during the analysis.

## Conclusion

There is an immediate impact on CFI change due to short-term educational intervention that improves infantile nutrition in rural Bangladeshi infants that can be generalized due to RCT design. This intervention strategy can possibly be adapted regularly at the national level. However, a large trial with multi-site diverse locations might still be required before routine intervention policy to improve under-five child’s nutrition and achieve the Sustainable Development Goal for resource-limited countries like Bangladesh.

### Generalizability

The CFI has been created through RCT, so its generated evidence is generalizable.

### Interpretation

The developed CFI indicates that short term nutritional educational intervention to mother and her family member has potentiality to improve her infant nutrition status.

### Registration

The registration number is NCT03024710 and date: 1/19/2017.

### Protocol

Can be accessed through: https://clinicaltrials.gov/ct2/show/NCT03024710

## Data Availability

Data contain potentially identifying or sensitive information from delivering women. However, “Data can be available on request”. The data request should be submitted to the Research Administration (RA) of iccdr, b and will be assessed by the corresponding Ethics committee named institutional Review 351 Board of icddr, b. As a supplementary information, we have added approved protocol where you can get the study title and protocol number (PR-17087) against which data access application should be made. Please visit https://www.icddrb.org/ dmdocuments/icddrb%20Data%20Access% 20Policy.pdf for additional information. Data requests are evaluated by icddr,b’s Data Repository Committee (DRC) and the Research Administration (RA) serves as the Secretariat of the DRC. The key contact person of RA at present is Ms. Armana Ahmed, Lead (A), RA at aahmed@icddrb.org If the data request is considered justifiable by the DRC then RA will share the anonymous data with the applicant. Moreover, for any particular clarification of the research findings that is documented in this article, queries can be directed to the primary author of this article or to the corresponding author. Both of them can be accessed at draminur@icddrb.org. The email correspondence regarding data access could be done at the executive director office at dircetor@icddrb.org.
